# Measuring the socio-economic and environmental outcomes of regenerative agriculture across spatio-temporal scales

**DOI:** 10.1098/rstb.2024.0157

**Published:** 2025-09-18

**Authors:** Katherine Berthon, Ruth Wade, Pippa Chapman, Coline C. Jaworski, Jonathan R. Leake, Niamh McHugh, Lisa Collins, Tim Daniell, Yu Zhao, Penelope Watt, Bob Doherty, Peter Jackson, Lynn V. Dicks

**Affiliations:** ^1^ Department of Zoology, University of Cambridge, Cambridge CB2 3EJ, UK; ^2^ Faculty of Biological Sciences, University of Leeds, Leeds LS2 9JT, UK; ^3^ School of Geography, University of Leeds, Leeds LS2 9JT, UK; ^4^ Université Côte d’Azur, INRAE, Sophia-Antipolis 06560, France; ^5^ School of Biosciences, The University of Sheffield, Sheffield S10 2TN, UK; ^6^ Game and Wildlife Conservation Trust, Fordingbridge, Hampshire SP6 1EF, UK; ^7^ School of Bioscience, University of Surrey, Guildford, Surrey GU2 7XH, UK; ^8^ Animal and Plant Sciences, The University of Sheffield, Sheffield S10 2TN, UK; ^9^ School for Business and Society, University of York, York YO10 5DD, UK; ^10^ Institute for Sustainable Food, The University of Sheffield, Sheffield S10 2TN, UK

**Keywords:** regenerative agriculture, co-design, soil health, systems approach

## Abstract

Regenerative agriculture aims to produce food while simultaneously improving soil health, supporting biodiversity, reducing input costs and enhancing climate resilience. Evidence on its environmental and socio-economic impacts across different systems and climates remains limited, with few studies measuring multiple outcomes following whole farming system transition. To be impactful, regenerative agriculture research must address farmers’ knowledge needs and provide practically feasible, economically viable solutions. This can be achieved through action-based research, co-designed with farmer stakeholders in real-world settings. Such research is time-consuming and involves potential risk for farmers adopting new practice combinations. Here, we describe two UK research projects gathering evidence on regenerative agriculture in partnership with farmers, at different scales. One is a replicated large-plot trial that stacks regenerative principles, the other a farmer-led quasi-experiment, following the transition in active farm businesses and using a flexible scoring system based on regenerative principles. We highlight benefits, challenges and future research directions emerging from these projects, including: challenges defining regenerative agriculture; co-design and maximizing knowledge exchange; generalizing results beyond study sites, when practices and outcomes are context-dependent; the need for interdisciplinarity; and generating evidence on long-term transitions with time lags between system change and outcomes, in an environment of short-term funding.

This article is part of the theme issue ‘Transforming terrestrial food systems for human and planetary health’.

## 1. Introduction

Agriculture accounts for 70% of land use in the UK and contributes to approximately 10% of the country’s total greenhouse gas (GHG) emissions [1]. Agriculture is also a leading cause of soil degradation, biodiversity loss, and pollution of both air and water [2], while being highly vulnerable to the impacts of climate change [3,4]. In addition, farm input costs have increased significantly in recent years owing to inflation and supply chain issues. For example, fertilizer prices in the UK have risen by over 40% since 2019 [5]. To reach net-zero GHG emissions by 2050, while also meeting food production needs and fulfilling environmental goals, a fundamental transformation of agricultural systems is necessary [6]. Regenerative agriculture, a farmer-led movement that aims to regenerate rather than degrade agricultural soils and ecosystems, is increasingly seen as a critical solution to the environmental, economic and social challenges facing modern farming [7,8].

Commonly, regenerative agriculture involves applying five principles—reduce soil disturbance, keep soil covered, maintain living roots, increase diversity and integrate livestock [9]—which guide the use of farming practices in a context-dependent way to achieve a variety of economic, social and environmental outcomes [10,11]. While multiple practices are associated with delivering these principles (table 1) [10,11,14,15], there remains considerable uncertainty in our understanding of the potential contribution regenerative agriculture can make to socio-economic and environmental outcomes across different farming systems and pedo-climates [15,16]. The uncertainty stems partly from the loose definition of regenerative agriculture [14,17], which allows farmers to adopt a spectrum of practices under the umbrella term [12], and makes it hard for those in other parts of the food system to be sure which farms are really ‘regenerative’.

**Table 1.** Principles, practices and potential outcomes of regenerative agriculture. Adapted from Giller *et al*. [10], Jaworski *et al*. [12], Jayasinghe *et al*. [11] and Voisin *et al.* [13].

principle	practices	desired outcomes
reduce soil disturbance	minimum tillage; zero tillage (direct drilling); controlled traffic; perennial crops including leys in arable rotations	reduced soil erosion; reduced loss of soil organic carbon; improved soil biology; increased water infiltration; reduced soil compaction; reduced costs (less fuel use); biodiversity conservation
keep soil covered	cover crops; retain crop residues; living mulch; leys, including herbal leys	soil carbon sequestration; improved soil structure; improved soil microbial activity; reduced soil erosion; reduced leaching of nitrogen into waterways; weed suppression; biodiversity conservation
increase diversity	crop diversification; leys, especially herbal leys; cover crops; intercropping; companion crops; mixed cultivar blends; agroforestry	biodiversity conservation; enhanced ecosystem services (biological pest control, pollination); improved soil fertility; improved soil biology; improved soil structure (different root structures); weed suppression; reduced synthetic inputs
maintain living roots	cover crops; leys/herbal leys; living mulch/understorey	reduced soil compaction; improved soil biology improved soil fertility; improved soil microbial activity; reduced soil erosion; improved soil structure (macroaggregates and macropores); increased soil organic carbon
integrate livestock	rotational grazing; manure and slurry applications	soil carbon sequestration; improved soil fertility; reduced synthetic inputs; improved soil biology

Many farmers are taking up practices and changing their farming systems to fit within a regenerative framework, but there is concern about the potential unintended consequences and trade-offs of these changes in the absence of evidence [12,18]. So far, incentives for regenerative agriculture adoption have focused on either the adoption of specific practices or the delivery of outcomes, with relatively little emphasis on the connection between them in different farming contexts [10,14]. For example, UK government incentives are rewarding the adoption of certain practices (e.g. cover crops, herbal leys, direct drilling) under the new sustainable farming incentive (SFI, [6]), while individual private supply chain schemes, e.g. McCains, Diageo and First Milk’s milk bonus payment [19] and collaborative public and private sector initiatives (e.g. Landscape Enterprise Networks (LENS) [20]) focus on outcome-based criteria, but give farmers control over the selection of practices used [21]. The lack of clear evidence-based best practice guidance and a framework that tracks the progress and effectiveness of regenerative agriculture leaves farmers uncertain about which practices they should implement to achieve their goals for their farm context. Without guidance, farmers risk making decisions that might not restore their systems efficiently or may even lead to unintended consequences or trade-offs, such as loss of yield, or enhanced pest problems. Lack of evidence-based best practice guidance, economic evidence and knowledge are commonly stated as barriers to the transition to regenerative agriculture [22].

Regenerative agriculture research is inherently complex owing to the vast variability between and within farming systems and combinations of practices being adopted. In addition, outcomes depend on numerous factors, such as different goals and approaches among farmers [23], including differences in experience and knowledge that lead to different interpretations of what regenerative agriculture systems are [18]; varied starting conditions, including different soil types, climates (e.g. [24]), farm history, size and type of farm business, and landscape context; and resource availability, such as equipment and inputs [25]. The outcomes of regenerative agriculture can also depend on the order, combination and timing of implementation of practices [26–28] and thus are affected by individual farmer decision-making.

Given these challenges, a system-based research approach is needed, where the outcomes are seen as emergent properties arising from the interactions of various regenerative agriculture practices [29]. Several previous studies demonstrate a systems-based approach using agricultural field experiments (see, for example, [30–34]), where the cropping system at plot or field scale is the unit of study, and the performance of different cropping systems is compared using multiple outcomes. Performance can be compared statistically based on either single or multiple outcomes, as long as sufficient replication at the system level is included in the design. Hawes *et al.* [31] analysed trade-offs between environmental and economic sustainability in an integrated arable cropping system. George *et al.* [33] studied intercropping with legumes to deliver sustainability through ecological principles. Hawes *et al.* [32] concluded that the scientific evidence on ecological approaches to farming, such as regenerative agriculture, is still limited, particularly our understanding of how best to combine practices for beneficial outcomes.

Despite this important work, there is currently a lack of information on the contextual suitability and practical constraints associated with adopting multiple regenerative practices. Recent syntheses of the impacts of regenerative agriculture on different outcomes are often collated in a practice-by-practice manner, from trials designed and conducted without the involvement of farmers, with the synergistic or antagonistic effects of implementing different combinations of practices rarely considered (but see [31,32,35]). There is an urgent need to develop a robust, evidence-based framework for benchmarking how ‘regenerative’ a farm is in terms of multiple outcomes and monitoring the impact of change on continuous gradients of system properties related to environmental, agronomic and socio-economic sustainability [36]. For such an assessment framework to be relevant at the farm level, any proposed indicator must (i) be cheap and easy to measure, (ii) relate to clearly defined outcomes, and (iii) be sensitive to management changes so that impact of a practice or multiple practices on an outcome can be predicted [36].

Here, we compare and contrast two ongoing co-designed research projects of the Transforming UK Food Systems (TUKFS) programme, FixOurFood and H3 (Healthy Soil, Healthy Food, Healthy People), both of which aim to gather evidence on the impacts of transitioning to regenerative agriculture on a range of environmental and socio-economic outcomes. Through comparison of these projects, we identify and discuss key challenges facing regenerative agriculture and its transformative potential for UK agriculture, including the challenge of categorization and definition of regenerative agriculture systems, benefits and challenges of co-design for regenerative agriculture research, the limitation of generalizing from single-site studies, the importance of interdisciplinarity for contextualizing ‘what works where’, and the challenge of conducting long-term research within short funding cycles.

## 2. The FixOurFood and H3 projects

The methods, benefits and challenges of the FixOurFood and H3 research projects on regenerative agriculture are summarized in table 2. The FixOurFood project co-designed and established a replicated large-plot trial at the University of Leeds farm in Tadcaster, Yorkshire, UK, in August 2022. Prior to the start of the project, the field was under 20 years of ‘conventional’ arable management, and the trial aims to:

— demonstrate and measure the effects of transitioning to different combinations of regenerative agriculture principles on soil structure and fertility, soil carbon, crop growth, development and yield, GHG emissions including carbon dioxide and nitrous oxide, above- and below-ground biodiversity, and profit to the farm business;
— understand which combinations of practices bring about the quickest and largest improvement in soil carbon, structure and fertility, support an increase in above- and below-ground biodiversity and reduce GHG emissions while maintaining crop productivity and farm profits.

**Table 2.** Comparison of the two different experimental approaches and their benefits and challenges.

	FixOurFood field trial approach	H3 farm cluster approach
experimental design	21 large plots, in a randomized block design: seven farming systems are each replicated three times across the field in three different blocks	quasi-experiment, where farmer-enrolled land parcels (‘blocks’) fit one of three categories: long-term regenerative agriculture, long-term conventional or transitioning farms
number of treatments	7	3
spatial scale	single field with multiple plots	farm blocks encompassing 1 to 10 fields
size of measurement area	three replicates of plots 12 m × 40 m = 480 m^2^ for each plot	4 or 5 replicates of 60 ha blocks for each treatment in each landscape (total 25 sites)
temporal scale (to date)	multi-year—min. of 4 years (longer-term dependent on continued funding)	multi-year—1 year baseline, 1 year changing practices, with 2 years of monitoring post-transition
stakeholder engagement	co-designed with local farmers and allied organizations, including agribusinesses, charitable organizations, government departments and agronomists	co-designed with local farmer clusters and allied organizations, including farm advisory groups, charitable organizations and agronomists
co-design approach	university determining trial design and implementing the practices based on local farmer advice and experience.	farmer-led decisions around implementation of new practices paired with academic monitoring of outcomes on farms
knowledge exchange design	trial has been designed to be a demonstration site for visitors to visually observe the differences between the systems and discuss the benefits and challenges of each system	experiment has been designed with farmer clusters, to build upon existing farmer knowledge exchange networks; results and monitoring tools (e.g. a regenerative agriculture score) are intended to be applicable across farms and distributed through stakeholder networks
location	University of Leeds farm, Tadcaster, Yorkshire, UK	two farmer clusters, one in east England and one in south England
soil type(s)	clay loam (42% sand, 28% silt and 30% clay)	loam to clay–loam; chalky silt to chalky clay
target metrics	— soil chemical, physical and biological properties — hydrological properties and water quality — soil-dwelling invertebrates (as indicators of soil health) — pests, disease and weed incidence — crop establishment, development, yield quantity and quality — costs and gross margins of each system — high-frequency N_2_O, CH_4_ and CO_2_ fluxes; C and N balances of each system — continuous site meteorological monitoring e.g. rainfall, soil temperature, solar radiation	— soil chemical, physical and biological properties — soil-dwelling invertebrates (as indicators of soil health) — biodiversity (birds, pests’ natural enemies, pollinators) and associated ecosystem services (pest control, pollination, soil properties) — pests, disease and weed incidence — yield and crop grain nutrient quality — farmer perspectives and political context — economic profitability — meteorological conditions (site temperature, humidity and wind speed)
benefits	— representative of real-world systems, with realistic, farmer-informed transition to regenerative agriculture — designed to experimentally test the impacts of stacking regenerative agriculture principles on outcomes in a replicated plot trial — detailed high-resolution data and measurements on multiple environmental outcomes and economics of each system — trial design and facilities at University of Leeds farm enables measurement of high-frequency GHG emissions of different farming systems — a demonstration site for visual comparison of different regenerative systems and knowledge exchange between stakeholders	— representative of real-world systems, with realistic, farmer-led transition to regenerative agriculture — designed to test applicability across two farming contexts — experiential learning—farmer decision-making involved in the trial (action-based research) — measuring outcomes at a spatio-temporal scale relevant to ecosystem processes and farmer decision-making — detailed reliable data on multiple environmental outcomes and economics — interdisciplinary (interactions between biotechnical and social dimensions of the experiment) — facilitates peer-to-peer learning within pre-existing farmer clusters
challenges	— measuring the impact of regenerative agriculture on one soil type and at one farm; requires additional trials on other soil types and farm contexts — farmers do not implement the practices on their own land as part of the experiment — challenges of deciding which practices to test; a large range of practices are associated with regenerative agriculture and there were multiple contrasting opinions among local farmers	— measuring the impact of regenerative agriculture on several major soil types; requires additional trials to test other soil types and farm contexts — less detailed information for any given context or practice combination — challenging to compare between sites as multiple variables covary across sites — challenges collecting practice data from farmers—making sure it is accurate and reliable — variation in the consistency and commitment to regenerative practices between farmers, and the impact of their decision-making on practice implementation

**Figure 1.**
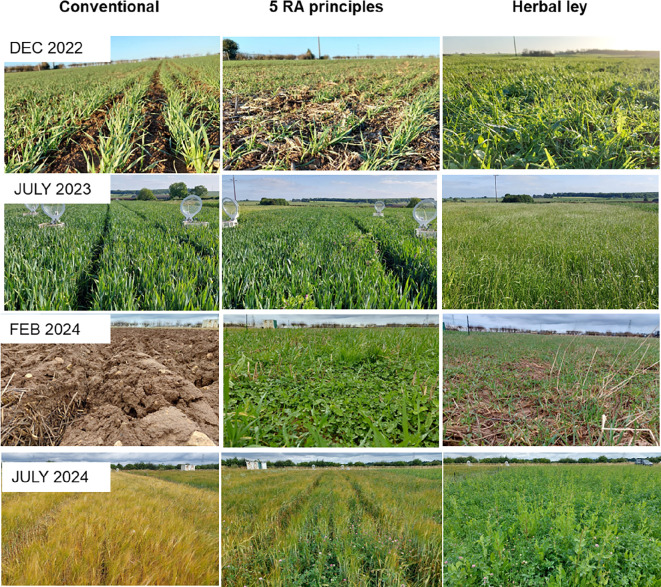
Photographs of three farming systems at different time points on the regenerative agriculture (RA) trial at the University of Leeds farm, Tadcaster, Yorkshire, UK: the ‘conventional’ system (left) (following no regenerative agriculture principles), the farming system following all five regenerative agriculture principles (middle), and a herbal ley (right) in December 2022, July 2023, February 2024 and July 2024.

The trial compares seven different farming systems by stacking regenerative agriculture principles from the most common to the least used principles (determined using data from a UK-wide survey [37] and personal communications at events and visits to farms), adding one additional principle until all five principles are implemented. The trial also includes two herbal ley treatments, which represent a different regenerative system where all five principles are followed but not in a cereal crop rotation for 3 years (figure 1). The seven farming systems follow the regenerative agriculture principles as follows:

(1) ‘conventional’: does not follow any of the principles by using practices such as inverted ploughing, leaving bare soil over winter, no livestock integration;
(2) two principles: (i) minimize soil disturbance and (ii) keep the soil covered;
(3) Three principles: (i) minimize soil disturbance, (ii) keep the soil covered, and (iii) increase crop diversity;
(4) four principles: (i) minimize soil disturbance, (ii) keep the soil covered, (iii) increase crop diversity, and (iv) integrate livestock;
(5) five principles: (i) minimize soil disturbance, (ii) keep the soil covered, (iii) increase crop diversity, (iv) integrate livestock, and (v) maintain year-round living roots;
(6) five principles: 3-year herbal ley with cereal rotation;
(7) five principles: long-term herbal ley without cereal rotation.

The trial consists of 21 plots, with each of the seven farming systems replicated in three blocks in a randomized block design (*n* = 3), to account for variation within the field and edging effects of the trial and treatments. The plots are large (12 × 40 m) to allow sheep grazing and the use of commercial equipment to reflect a real farm system, with areas for destructive and non-destructive measurements. The practices followed within each principle for each treatment are shown in figure 1 and table 3.

**Table 3.** Practices implemented under different principles followed in the regenerative agriculture trial at the University of Leeds farm, Tadcaster, Yorkshire, UK. Boxes with italicized text show the practices where the principle is being followed, whereas plain text shows the practices where the principle is not being followed.

		regenerative agriculture principles				
treatment no.	no. of principles	minimize soil disturbance	keep soil covered	increase diversity	integrate livestock	keep a living root all year
1	none	ploughing and power harrow between crops	soil left bare over winter between crops	single variety cash crops	none	annual crops
2	2	*where possible, direct drill; otherwise minimal shallow cultivation*	*cover crops over winter*	*5-species cover crop and single variety cash crops*	none	annual crops
3	3	*where possible, direct drill; otherwise minimal shallow cultivation*	*cover crops over winter*	*10-species cover crop and mixed variety cash crops*	none	annual crops
4	4	*where possible, direct drill; otherwise minimal, shallow cultivation*	*cover crops over winter*	*10-species cover crop and mixed variety cash crops*	*sheep grazing winter wheat early spring and cover crops; addition of pig straw and muck*	annual crops
5	5	*where possible, direct drill; otherwise minimal, non-inverted cultivation*	*living mulch/understorey growing all year*	*5-species mix understorey/living mulch and mixed variety cash crops*	*sheep grazing winter wheat early spring and living mulch; addition of pig straw and muck*	*perennial living mulch/understorey*
6	5: 3-year herbal ley with cereal rotation	*no soil disturbance for 3 years while herbal ley*	*herbal ley growing for 3 years*	*18-species mix: GS4 herbal ley mix from Cotswolds Seeds*	*sheep grazing herbal ley*	*herbal ley growing for 3 years*
7	5: long-term herbal ley (no cereal)	*no soil disturbance*	*permanent herbal ley*	*18-species mix: GS4 herbal ley mix from Cotswolds Seeds*	*sheep grazing herbal ley*	*permanent herbal ley*

The H3 project is investigating implementation of regenerative agriculture on commercial farms, using a quasi-experimental approach where farmers make decisions about best use of practices for their farming system in a transition to regenerative agriculture, but within the framework of a before–after control–impact (BACI) design [38]. This approach allows inferences to be made about the effect of changing practices [39], while recognizing the spectrum of regenerative practices adopted across fields within and between farms. The research methods are described in detail elsewhere [38], but, in short, the experiment involves 60 ha blocks of land that farmers volunteered to manage in one of three ways: (i) adopt new regenerative practices as part of the project from 2022 (transition farms), (ii) farmers that have been using regenerative practices for at least the last 3 years will continue to do so (regenerative farms), and (iii) limit their use of regenerative practices during the study period (control/conventional farms). The H3 experiment spans two UK landscapes, one clay soil arable and a second chalk soil mixed arable, with four farm blocks per group in each landscape. Each farm is monitored for biodiversity (represented by birds, natural enemies of crop pests, and pollinators), soil physical, chemical and biological properties, and yield and crop quality across three harvest seasons.

**Figure 2.**
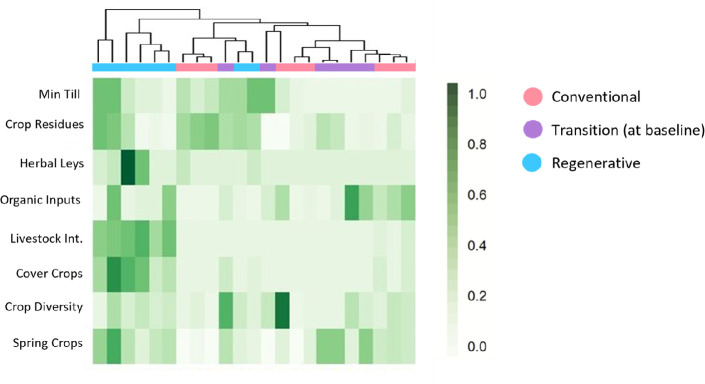
The ‘fingerprint’ of the most commonly implemented regenerative practices on H3 farms from 2018 to 2022 (baseline, before transition). Each column represents an individual farm, and the shading of the squares represents the consistency with which regenerative practices (rows) have been implemented over the past 5 years (max. 1 = implemented every year). The colours in the cluster diagram represent the status of the farm site as enrolled by farmers in 2022 (i.e. transition farms had not transitioned yet).

At the beginning of the project, nine agricultural practices were chosen for which there was agreement across both farming landscapes and with scientists (based on scientific evidence) that they matched regenerative principles for their farming context, and that transition farmers were willing to adopt. These were: no or minimum tillage, retention of crop residues, use of cover crops (including ‘catch’ crops sown before winter crops), spring cropping, herbal leys, reduced soil compaction techniques, organic matter addition, livestock grazing and crop diversification. However, the flexibility in the definition of what it means to be regenerative by farmers in the project [18] has resulted in different baselines for each farm and each farmer cluster in their application of regenerative practices (figure 2). There is a gradient of practice implementation; not all regenerative farms are implementing all regenerative practices, with ploughing still occasionally used to move from leys to other crops, and many of the control farms already implement some regenerative practices, such as retention of crop residues and controlled traffic.

## 3. Challenges of quantifying the benefits or disservices of regenerative agriculture

### (a) The categorization problem

The complexity of regenerative agriculture transitions makes it difficult to categorize regenerative systems and measure the cumulative and synergistic or antagonistic effects of adopting multiple regenerative practices on different outcomes. Multiple practices can be implemented to achieve regenerative principles (table 1) and variation in the outcomes of regenerative practices may lead to application of different practices to reach a similar goal in different contexts. For example, multiple regenerative practices have been shown to result in soil carbon sequestration [15], but the magnitude of soil carbon sequestration varies depending on soil type and climate [24,40]. Several regenerative practices are rotational, being applied on one or a few fields per year. For example, many farmers apply farm yard manure (FYM), herbal leys or cover crops to one or a few fields at a specific stage in the crop rotation. Consequently, the transition to regenerative agriculture is often measured as the implementation of regenerative practices, rather than quantifying effective implementation of regenerative principles on a yearly or short-term basis.

Several methods for categorizing regenerative systems based on the adoption of practices have been proposed in the literature. Most studies define a binary categorization of regenerative agriculture through a threshold process, where farms achieve regenerative status through a minimum implementation of practices (e.g. [32,41]), while others present the gradient of implementation using the cumulative inclusion of practices (e.g. [42]) or a classification based on different combinations of practices, and the relative contribution of practices to the regenerative principles [12]. However, neither cumulative addition nor binary use/exclusion (presence/absence frameworks) of practices considers variation across time, or consistency in the application of practices across fields. The outcomes of implementing regenerative agriculture can be measured at different spatial and temporal scales, and assessment must take into account context dependencies related to different soil types, climate and farm systems [10]. When using practice-based definitions, matching practices to principles goes part way to acknowledging the mechanisms underlying contribution of regenerative practices to regenerative outcomes [12], but needs to be scaled relative to how frequently a practice can be implemented across fields, and how well the practices achieve the desired outcomes for a given context.

The FixOurFood project has tackled the problem of categorization through the experimental ‘stacking’ of regenerative principles and choosing regenerative practices that can achieve these principles for their specific soil type, through co-design with local farmers (table 3). These categories were decided based on a survey of 130 UK farmers as well as individual phone and in-person discussions and on-farm tours, to determine which principles and practices were most commonly being used by regenerative farmers [37]. This categorization allows testing of five different regenerative systems, and the effectiveness of different combinations of regenerative principles as well as practices. Management decisions within each system are taken based on the combination of principles being used, and the experience of local farmers. The data derived from this replicated trial will be an important resource for farmers and policymakers in guiding decisions on how to modify existing agricultural systems to reconcile multiple objectives (economic, including food production, ecological, environmental).

**Figure 3.**
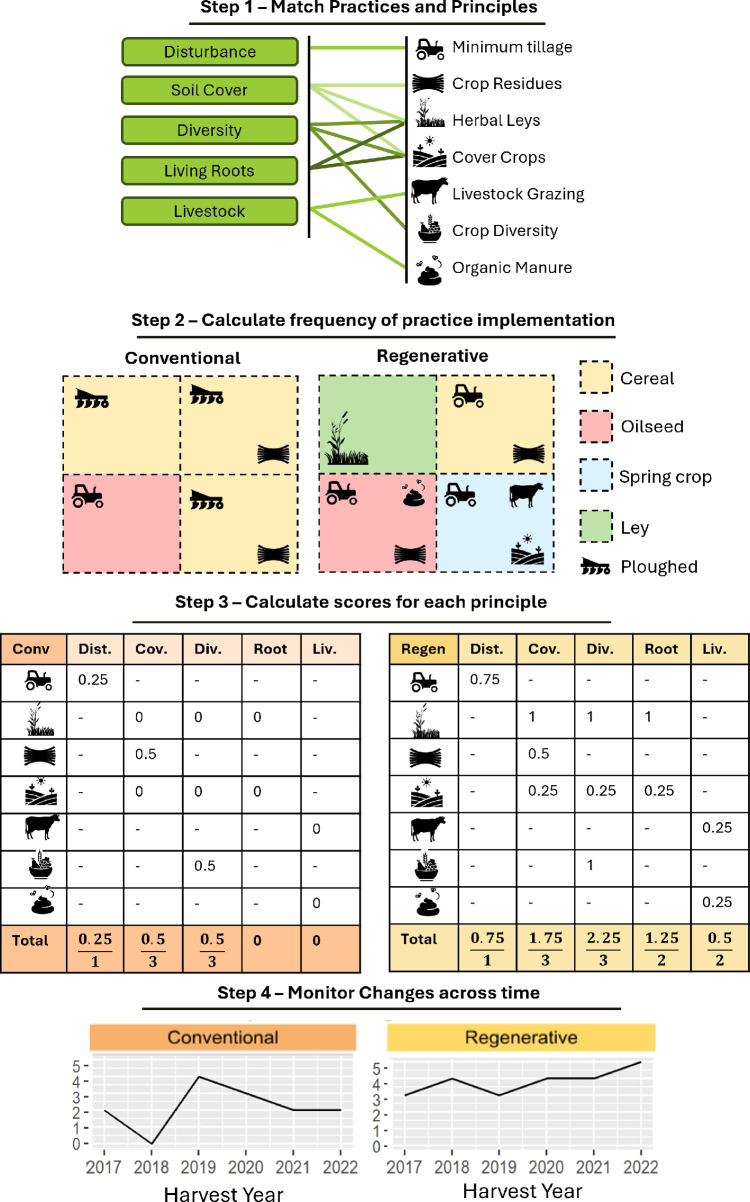
An application of the H3 scoring system to two farms, one conventional (Conv) and one ‘regenerative’ (Regen) farm. *Step 1***—**once practices are defined for a farming context, they are matched to the relevant regenerative principles. *Step 2*—the relative frequency of each practice in a given year is determined from the proportion of the farm on which it is employed, relative to the maximum area available. For example, herbal leys are only present in one or a few fields per year and are given a binary score. Similarly, crop diversity is scaled relative to the number of fields present so as to not penalize smaller farms. *Step 3*—the relative principle scores are generated based on the average of the scores for relevant practices, giving a possible total of 1 for each principle. Dist. = minimize disturbance; Cov. = maximize cover; Div. = diversity; Liv. = integrate livestock. *Step 4*—monitor changes across time by building a cumulative score across principles that can later be matched with outcomes of interest (figure 4). For more detailed information on the mathematical underpinnings of the scoring system, please see the electronic supplementary material.

**Figure 4.**
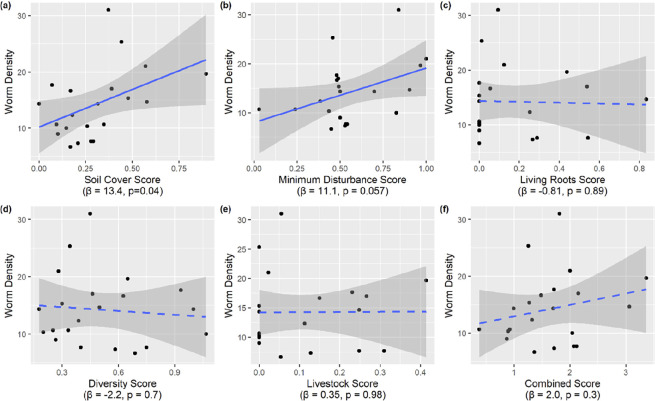
Application of the H3 scoring system to test the impact of implementing different practice combinations on earthworm density (average worms/site). Individual practice scores for (a) soil cover and (b) minimizing disturbance have a larger impact on earthworm density, than individual scores for (c) maintaining living roots, (d) increase diversity and (e) integrating livestock. Overall scores aggregated across principles (f) explain variation in earthworm density less well than some of the individual principle scores. Dashed lines indicate non-significant trends. See electronic supplementary material for more detail on model outputs.

Alternatively, the H3 project has developed a multivariate method to fingerprint each farm (figure 2) using the consistency of regenerative practices as implemented across the previous 5 years. Similar to Jaworski *et al.* [12], each practice is matched to the related regenerative principle(s) it achieves and farmers can use this information to provide a relative ‘score’ that tracks their progress in practice implementation over time (figure 3). Scoring reflects the implementation of each regenerative principle and the consistency and efficacy with which the implemented practices meet the regenerative principles. In this way, a separate score is generated for each principle from the combined frequency of its constituent practices across time. These principle scores can then be used to represent the degree to which all five principles are being fulfilled on a farm. For illustration, figure 3 demonstrates the use of the regenerative score on two hypothetical farms, one regenerative and one conventional, using a subset of the practices given in table 1. Figure 4 shows an application of this to the H3 dataset, and more detailed description of the mathematics behind the scoring system is given in the electronic supplementary material.

The H3 regenerative agriculture scoring system is transferable to different farming contexts, by including or excluding practices based on contextual suitability and future innovations. For example, living mulches and intercropping were not agreed core practices for the H3 project but could be relevant as part of regenerative farming systems in other contexts. Similarly, soil health indicators for organic-rich lowland peat soils are very different from those for mineral soils [43] and may require modified principle and practice matches (Farmers, personal communications). However, the choice of practice or principle inclusion must take account of existing scientific evidence for efficacy, as well as practicality in context, and exclusion should not be solely based on farmer choice, to avoid bias in farmers' perceptions of their sustainability or fit [12]. The selected practices are expected to deliver the outcomes through meeting regenerative principles, but the magnitude of the response is likely to vary across farms in different contexts. Further development of this scoring system would add weight to practices or principles based on the magnitude of their expected impact on the outcomes of interest in a given context. For example, the impacts of cover crops on soil properties depend on planting date, plant species used and biomass produced [44].

Importantly, this scoring system does not give an indication of the scale or extent of regenerative outcomes (e.g. the extent of improvements in soil and biodiversity) but rather is a standardized way of comparing how consistently regenerative practices are performed. The H3 experiment will use the scoring system to assess the impact of transitioning to regenerative practices, by correlating changes in the relative farm scores with changes in environmental outcomes for biodiversity and soil health. This can be achieved by aggregating the principles into a unified score across farming seasons, or by relating individual principle scores to outcomes (figure 4). While the former may be more useful from a policy or certification standpoint, it is likely that the latter is more informative from a mechanistic perspective as some principles are more relevant to achieving certain outcomes. For example, cover crops have limited impact on carbon sequestration [15] but can substantially decrease soil erosion and leaching of nutrients into waterways [44]. Similarly, earthworm density on H3 farms in the baseline year was better predicted by 5-year averages of the ‘maintain soil cover’ (*β* = 13.4, *p* = 0.04) and ‘minimize soil disturbance’ (*β* = 11.1, *p* = 0.057) scores compared with a combined score (*β* = 2.0, *p* = 0.3; figure 4). Methods for collection of earthworm data are given in [38]. In this example, more recent implementation of the regenerative practices is weighted higher than previous years, to allow for cumulative impacts of regenerative practices over time. For example, soil health shows continuing improvement after multiple consecutive years of no-till [45].

### (b) Co-design, engagement with farmers and knowledge exchange opportunities of regenerative agriculture research trials

Since being ‘regenerative’ can encompass a range of aims, practices and outcomes that mean different things to different stakeholders [8,18], what ‘success’ looks like may also vary between farmers and researchers [46]. Therefore, engaging with farmers, and integrating diverse stakeholder perspectives in co-designing, co-creating and co-producing scientific knowledge and evidence to inform regenerative farming systems and demonstrate their economic, environmental and social potential will be pivotal in scaling the transition to regenerative agriculture effectively and sustainably. Both projects took an action-based research approach, with co-design and co-production of knowledge to ensure that the research is impactful to the farming community and that the on-farm trials are scientifically valid [29,46,47], address farmers’ and other stakeholders’ questions and evidence requirements, and present solutions that are practically achievable [10].

The two projects involved farmers in the decision-making and research process in different ways, resulting in different modes of learning and knowledge exchange (table 2). FixOurFood co-designed the agricultural field experiments at the University of Leeds farm in collaboration with local farmers and allied stakeholders, and the trial acts as a demonstration site comparing seven different farming systems at a single site, with free tours and workshops to encourage farmer-to-farmer and farmer-to-researcher knowledge exchange and learning. This allows farmers access to view and discuss the practical experience of implementing new practices, but researchers maintain control over the management decisions, the practices implemented within each farming system and the measurement and quantification of economic and environmental outcomes. Farmers can use learning from the demonstration site to explore options for their own farms, evaluating the pros and cons of different combinations of principles and practices, hearing about best practice and outcomes before committing to implementing them on their farm, and thus reducing risk to their business. As the different systems are demonstrated next to one another, it is easy to visibly see the different systems and discuss their benefits and challenges. Demonstration sites like these are well known tools to increase social learning [48] and encourage adoption of agricultural practices [49]. While farmers can easily come and view the demonstration trial, they are at different points in their regenerative agriculture journey, with some having transitioned several years ago, some just beginning their journey and others interested in transitioning but not yet having taken the first steps. Thus, what they take away from their visit to the trial varies and the impact of the trial in changing farmers' behaviour has not yet been tracked.

The H3 project was co-designed through collaboration with farmer clusters, collectives of farmers already working together within landscapes [50]. Farmers transitioning to regenerative agriculture in the H3 clusters experience first-hand its challenges and opportunities in their context and learn by actively engaging in adopting new practices on their own land. This is known as experiential learning, which is the process of learning by doing [51]. Farmers also learn via peer-to-peer learning within the farmer clusters, through a series of discussion groups, farm-walks and workshops, organized by farmer cluster facilitators. Peer-to-peer networks have a large influence on farmer decision-making, and can encourage the adoption of new practices [52], by both participating farmers and their broader networks [53]. However, on-farm trials pose economic risks [22] and high uncertainty [54], and involve significant time investment from farmers. The H3 project has provided some monetary support for farmers transitioning to regenerative agriculture to help reduce economic barriers to uptake and mitigate some of these risks. These risks are higher for tenant farmers, who rely on the economic profitability of their businesses [55]. Therefore, farmers who enrol in trials like the H3 one, are more likely to be conservation-minded, and engage in regenerative practices as a baseline, generating potential biases in the selection of study sites [49].

Transitioning to regenerative agriculture requires transformational changes in practices, but also potentially in farmers’ attitudes and beliefs [23,56]. Direct engagement of farmers in co-producing knowledge about what works is more likely to lead to long-term adoption of regenerative practices [46,53]. However, demonstrations and on-farm experiments are also most successful when adapted for local contexts, providing locally relevant and soil-specific information to farmers [29]. There is a need to engage with stakeholders in policy and industry to devise enabling practices, policies and legislation that stimulate the uptake of regenerative agriculture [57]. Therefore, future projects on regenerative agriculture need to continue to adopt co-design approaches, but in different farming contexts, extending the work of the FixOurFood and H3 projects.

### (c) Generalization issues: scaling up from single sites to landscape-scale approaches

The FixOurFood experiment will provide direct causal evidence for the effect of implementing practices associated with between two and five stacked regenerative principles, with a known cropping history, a unified starting soil condition and each farming system replicated three times. The scale of the trial allows detailed and frequent measurements of environmental outcomes over multiple years. This kind of time-series data enables elucidation of causal relationships between practices and outcomes that regenerative principles seek to achieve [39]. The project has focused on stacking principles that represent the decision-making process of farmers transitioning to regenerative agriculture, on the basis of the initial surveys, which showed the most common combination of principles is keeping the soil covered and minimizing soil disturbance [37]. Whilst the trial enables robust replicated evidence of the impact of stacking regenerative principles on multiple ecosystem services, it is only providing evidence on one farm, on one soil type and with a specific climate. This limits applicability of the findings to other contexts with different soil types. There is significant opportunity to utilize this experiment as a detailed trial site, where practice combinations that bring about the greatest regenerative impacts on this site are then trialled on other ‘satellite’ farms. This would enable new trials to focus experiments on specific practices and measurements of key outcomes rather than needing all practice options trialled and all measurements taken at every site/location.

The H3 project does not have the same level of direct control over practice combinations being used by farmers or starting soil condition and legacy of the fields in its study. Every field has been managed uniquely by the combined knowledge and experience of individual farmers and their agronomists, and many factors are changing simultaneously [38]. As a result, the study design violates several assumptions necessary for causal inference [58], in particular ‘excludability’ (because the process by which treatments were assigned could affect the outcomes), ‘no interference between sampling units’ (because farmers are encouraged to learn from each other), and ‘no multiple treatment versions’ (because not all farmers stuck rigidly to the assigned treatments). This reduces the external validity of the study, and its ability to infer causation, but these shortcomings are hard to avoid in genuinely co-designed, action-based research. Nonetheless, the sampled blocks are matched in terms of size, farming system and soil type, and involve multiple replicates in each landscape, which allows relative comparisons of these unique farming approaches [39]. This limitation in uniformity (unique farm trajectories) is partly balanced by a larger sampling size and the larger spatial scale, which allows measurement of ecosystem processes that work at this scale (e.g. biodiversity conservation). We consider the H3 experiment to have some potential to generalize beyond its specific contexts, because results across multiple farms, in different landscapes on a range of soil types, and using similar approaches, are more likely to hold on other farms in different contexts [29].

The distinct approaches from these two experiments bring complementary evidence. The direct mechanistic understanding from FixOurFood will inform the interpretation of results across farms in the H3 project. Detailed measures of regenerative outcomes from different practice combinations can also directly feed into the weighting of different principles and practices in the H3 scoring framework, and work towards application of the scoring system across different farming contexts. Additionally, the H3 project could extrapolate whether the practice combinations used in the FixOurFood experiment show similar results when applied across multiple fields, on farms with different farming histories, equipment and soil conditions.

Ideally, this would be a nested design, where the FixOurFood experiment would be embedded in one of the H3 landscapes so the results were directly transferable between projects. This kind of ‘hub and spoke’ approach would be beneficial for integrating insights across different farming contexts; however, the nature of disparate and short-term funding makes this kind of co-ordinated approach difficult (see §3e). Several long-term experiments investigating farming systems in the UK already exist (e.g. [34]), but significant added value could be gained by collaborating across these sites using common, standardized protocols for sample collection and data analysis procedures. There are also international examples of co-ordination of networks at a cross-country scale (e.g. the eLTER initiative, https://elter-ri.eu), which brings together research centres to study the long-term effects of environmental, societal and economic factors on ecosystems. For regenerative agriculture research, a first step would be a platform for data-sharing that compiles the impacts of combinations of practices across projects, as well as integrating farmer insights.

### (d) The need for interdisciplinarity: integrating evidence across multiple outcomes

The successful transition to regenerative agriculture involves adoption of regenerative farming practices to achieve several and often conflicting goals [10]. There are trade-offs in the efficacy with which practices achieve these different goals [59], and how we measure success of the transition to regenerative agriculture will depend on the goals of implementation. Therefore, there is a need to combine a range of different metrics, across different disciplines, to measure the multiple outcomes from implementing regenerative practices and how they vary from one field/farm to another. A specific challenge for regenerative agriculture research is to define appropriate metrics that can be standardized across such diversity while being highly informative for measuring progress towards outcomes, relevant to and used by farmers in the context of a flexible definition of regenerative agriculture [29,59].

Given universal limitations in evaluation capacity, there are inherent trade-offs between resolution of information, and the breadth and scale at which measurements can be made. Both research approaches of FixOurFood and H3 enable the measurement of a wide range of ecosystem services but at different spatial and temporal scales. FixOurFood has high-resolution data over a smaller spatial scale, with repeat measurements of GHG fluxes every 120 min, soil pore water sampled weekly when soil moisture allows, and soil sampling taken at 10 cm depth intervals to bedrock after each crop. This resolution is needed when equipment limitations require smaller scales (e.g. the GHG Eosense chambers have a spatial measurement limit of 20 m from the Picarro analyser and can support a maximum of 12 chambers) and for understanding the immediate impacts of practice changes on soil structure and quality within fields, but limits exploration of ecosystem processes at a broader scale, e.g. biodiversity. Conversely, the H3 project covers a much larger area, but measurements are taken at a lower resolution, with outcomes determined on one to three fields per farm per year [38]. This gives a much coarser picture of the change in outcomes over time, but over a scale that encompasses ecological processes such as changes in biodiversity and related ecosystem services, particularly for species with large home ranges such as birds and pollinators.

Importantly, environmental outcomes are measured simultaneously to economic outcomes in both projects, to understand the ability of regenerative systems to achieve food production *and* protect the environment [10]. Social perceptions and farmer motivations have been captured, but at different scales across the two projects. The FixOurFood project began with a quantitative survey that assessed farmer perceptions, implementation and perceived barriers to uptake of regenerative practices across the UK, to inform their trial designs [37]. The H3 project took a qualitative approach to understanding farmer perspectives on the definition of regenerative agriculture, and their motivations for and perceived barriers to uptake of regenerative practices [18]; the project then follows the journey of these same farmers as they adopt new practices and navigate a shifting policy landscape [38]. The project is complementing this analysis with a series of interviews with policymakers, to understand how regenerative agriculture can be better integrated into new policy frameworks.

### (e) Challenges of short-term funding to evaluate long-term changes

Regenerative systems can be based on crop rotations that can last 10 years or more, and include a diversity of crops and practices. Many regenerative outcomes are only achieved after long-term implementation of regenerative practices, with a possibility of opposite effects, such as yield losses in the early years [9]. In the UK, research projects are typically funded for 1−5 years, not providing enough time to fully measure and realize the potential of regenerative agriculture in different years (when weather patterns may differ) and across diverse rotations. Research also often occurs in larger consortium projects such as those funded by the Transforming UK Food Systems programme, where regenerative agriculture is only a relatively small part of a larger set of research objectives. Such projects are quite disconnected and would benefit from integration, notably for synthesizing farmers’ knowledge, and integrating co-designed experimental approaches across projects and geographical areas [57].

Furthermore, co-designed research requires the development of trust between farmers and researchers, and a lot of effort and work goes into establishing and maintaining these relationships during projects. Once projects end, it can be difficult to keep momentum and engagement going, without ongoing financial support. Advisory groups like the Farming & Wildlife Advisory Group (FWAG), organizations with an advisory arm (e.g. Game and Wildlife Conservation Trust), farmer-led organizations (e.g. Nature Friendly Farmer Network, LEAF and Agricology) and farmer cluster facilitators provide some of this structure to aid knowledge-sharing among farmers. There needs to be an equivalent commitment from the research community in building ongoing infrastructure for community exchange to support farmer decision-making, evidence gathering and synthesis over the long term [34,57]. This demands longer-term funding mechanisms and nationally co-ordinated research infrastructure, to support on-farm trials and knowledge exchange hubs that can inform an evidence-based transition to regenerative agriculture.

## 4. Future research

Given the scale of the challenges facing UK agriculture, the range of farming and soil types across the UK and the radical transformations taking place in government support for more environmentally friendly farming, there is a need for ambitious national-scale efforts to synthesize farmers’ knowledge of regenerative agriculture and align on-farm research projects. Additional regenerative agriculture trials are needed to investigate innovative combinations of practices that are currently underexplored but offer exciting potential to deliver regenerative agriculture goals. Suggested future research priorities include investigating new ways to terminate leys and cover crops and control weeds, more data on GHG fluxes and biodiversity outcomes, improved contextual understanding of the pros and cons of including livestock into regenerative systems, improved understanding of the challenges of transitioning to regenerative agriculture, modelling the impact of a range of climate change scenarios on regenerative agriculture outcomes, and expanding farmer participation to encompass varied agricultural settings. In particular, the financial viability of regenerative agriculture needs to be evidenced, particularly in the early stages of transition, to assess the need for financial support mechanisms to de-risk and accelerate the transition.

Together, the FixOurFood and H3 projects can provide evidence on the scale and extent of outcomes achieved by implementing regenerative practices that are commonly used across three farming areas of the UK. These projects are beginning to answer outstanding mechanistic questions about the effects of different regenerative practices, and how outcomes develop over time. Demonstration sites like the FixOurFood field trial are crucial because they provide a practical, visible way to showcase the benefits, feasibility, and outcomes of specific practices or innovations, helping to bridge the gap between theory and application [60]. Similarly, landscape-scale experiments like the H3 project are essential to address the complexity of agroecological processes, capture interactions across multiple scales, and develop integrated solutions for sustainable agricultural systems in heterogeneous landscapes [61].

There is a need for more context-specific, or externally valid, information to ensure the results are applicable to other farming systems in the UK and across Europe. This can be achieved through engagement with farmer clusters and establishment of a network of regional research hubs that cover typical soil types/climates in the main UK cropping regions. These hubs could be strategically established in contrasting contexts to encompass variation in soil composition, levels of soil degradation, differences in typical practice combinations between landscapes, and socio-economic factors, to increase feasibility given the sheer number of theoretical practice combinations. Such hubs would enable smaller-scale, targeted research trials based on evidence from the hub, as well as large-scale coordinated trials of the same practice combinations across different contexts. Most farmers constantly engage in on-farm experimentation themselves and have in-depth knowledge of what works and has not worked for their farming context. This information can be integrated with research findings to develop tools for farmers to track their progress in implementing regenerative agriculture (e.g. the H3 scoring system).

Specifically, we call for a coordinated, national regenerative agriculture research programme across universities and farming organizations, including creation of a platform with synthesis of regenerative agriculture research accessible to farmers, updated annually. There are significant opportunities to improve the research infrastructure to foster collaboration between research projects, farmers and allied stakeholders to improve understanding and quantification of the long-term impacts of regenerative agriculture on socioeconomic and environmental outcomes. Metrics to track the progress and effectiveness of regenerative agriculture are underdeveloped, as is the evidence needed to guide farmers and policymakers as to which regenerative practices to adopt or support.

## 5. Conclusions

This perspective gives an overview of two ongoing complementary co-designed applied research projects that aim to improve our understanding of the impacts of regenerative agriculture on soil health, biodiversity, GHG emissions, crop production, and farm profitability: the FixOurFood plot-trials at the University of Leeds farm, and the H3 quasi-experiment on commercial farms, in collaboration with farmer clusters in the south and east of England. Both projects have been co-designed with farmers to produce context-specific data on environmental and socio-economic outcomes from whole-system transitions to regenerative agriculture. The detailed measurements of field-scale outcomes can be paired with the utility of a scoring system to track implementation, offering insights to inform regenerative agriculture adoption and policy frameworks.

The paper has identified some of the key challenges involved in understanding the outcomes of regenerative transitions, including: the resources required for genuine co-design with farmers and allied stakeholders, how to maximize the knowledge exchange opportunities from regenerative agriculture research trials, generalizing results from single study sites, the challenge of context (what works where), the challenges of short-term funding when aiming to build an evidence base that supports longer-term transition to regenerative agriculture and the need to engage with allied stakeholders (policymakers and industry partners) to ensure enabling industry practices, policies and legislation that stimulate the uptake of regenerative agriculture.

## Data Availability

Detailed methodology and calculations are provided in the online supplementary material. Data are also provided in the supplementary material as is the code to make the figures [62].
